# Evaluating the use of seaweed extracts against root knot nematodes: A meta-analytic approach

**DOI:** 10.1016/j.apsoil.2021.104170

**Published:** 2021-12

**Authors:** Tamsin I. Williams, Steve Edgington, Andy Owen, Alan C. Gange

**Affiliations:** a Department of Biological Sciences, Royal Holloway University of London, Egham, Surrey TW20 0EX, UK; b CABI, Bakeham Lane, Egham, Surrey TW20 9TY, UK; c ICL, Koeweistraat 4, 4181CD Waadenburg, the Netherlands

**Keywords:** PPN, plant parasitic nematode, RKN, root knot nematode, Seaweed, *Meloidogyne*, Effect size, IPM, Nematode abundance

## Abstract

Plant parasitic nematode (PPN) control has historically relied on the use of synthetic chemical nematicides, however many are toxic to both human health and the environment. The withdrawal of the more harmful nematicides coupled with increases in soil temperatures and increased occurrence of pests and diseases associated with climate change, may enable PPN to increase in numbers and spread globally. The need for sustainable and environmentally friendly management options is necessary while facing future food security scares in order to feed the ever-growing population. Seaweed extracts have been used for decades in agriculture and horticulture as soil biostimulants, however there is a growing body of evidence to suggest that they could be used to reduce the occurrence of damaging PPN infections. Using meta-analysis, we investigated whether seaweed extracts applied to soil could reduce root knot nematode (RKN) abundance and whether there could be confounding factors that influence their efficacy. We found that seaweed extracts reduce RKN performance and that various factors affected the efficacy of seaweed, including the seaweed species itself and the crop the seaweed was applied to. *Ascophyllum nodosum* extracts were found to be the most effective. Particular RKN species were more sensitive than others to seaweed species used and, in some cases, specific seaweed species only affected particular RKN species*.* Different life cycle stages were also differentially susceptible to seaweed application, where both egg hatching and population abundance could be reduced via seaweed use. This research indicates that seaweed extracts could potentially be used to help reduce RKN attack on plants.

## Highlights

• We show that seaweed extracts can reduce root knot nematode performance.
• Nematode species respond differently.
• Some seaweed species are more effective than others.
• Seaweed efficacy differs between plant species.

## 1 Introduction

Plant parasitic nematodes (PPN) are microscopic soil-borne worm-like parasites that cause crop devastation and threaten global food security annually. Around 4300 species of PPN have been identified (Decraemer and Hunt, 2013) attacking a wide range of crops. Many PPN produce generalised symptoms above-ground, e.g. wilting and yellowing of the plant, which makes diagnosis difficult for farmers. Crop losses caused by PPN are in the billions (US $) and they are particularly devastating in tropical and sub-tropical regions (Abad et al., 2008). Some estimate that global crop losses due to nematodes are above 1% for some crops. For example 9.3% of global soybean yield loss is attributed to the cyst nematode and root knot nematodes (RKN) may cause an additional 1% of global soybean crop losses (Savary et al., 2019). However, afflicted crops are not limited to those in the tropics as PPN damage can also devastate a wide range of temperate crops, including cereals, *Solanum lycopersicum* (tomato) and *Solanum tuberosum* (potato), as well as long-term grass production in amenity and sports turf (Allan-Perkins et al., 2017; Peiris et al., 2020).

PPN control has relied heavily on the use of synthetic chemical nematicides in the past, using active ingredients such as carbofuran and methyl bromide, however a number of these chemicals have been withdrawn from the commercial market as they pose a danger to both the environment and human health. Chemical nematicides often are highly toxic to mammals and other non-target organisms (Haydock et al., 2013). For this reason there is an increasing search to find ‘natural’ nematicides and consider integrated pest management approaches (Peiris et al., 2020; Waisen et al., 2020). This is of particular importance when facing global temperature rises as increases in soil temperatures allow faster multiplication of many PPN, increasing population booms and higher levels of plant damage. Furthermore, prevalent PPN genera that currently cause only minor damage may become the pests of the future, for example in the UK it has been predicted that warmer soil temperatures may benefit the potato cyst nematode *Globdera rorostochiensis*, which is currently only a minor pest in southern parts of the country (Jones et al., 2017). We may also see a greater global spread of PPN, where predominately tropical PPNs may move to the warming temperate regions (Somsekhar and Prasad, 2012).

It is only a small number of nematode genera that are of particular economic importance and species of *Meloidogyne*, *Pratylenchus* and the cyst nematodes (*Heterodera* and *Globodera* spp.) cause the most damage (Jones et al., 2013). These nematodes differ in that some are generally polyphagous (*Meloidogyne* spp.) whereas others such as *Heterodera glycines*, are monophagous and only attack soybean crops (Hassan et al., 2013). Root knot nematodes (RKN) are one of the most researched PPN due to their economic importance and global spread. RKN are obligate sedentary endoparasites of plants. While above ground symptoms caused by RKN may be generic, below ground symptoms are distinctive, as infection causes root galling. Root knot nematodes parasitise a wide range of plants and can thrive in a wide variety of climatic zones from temperate to tropical (Álvarez-Ortega et al., 2019).

A sustainable and eco-friendly method of managing PPN populations (and more specifically RKN populations) that has recently gained attention is the application of seaweed extracts to crops and soils. Sustainably-sourced seaweed extracts have been used for decades as fertilizers and soil conditioners. They have been used primarily to stimulate root and shoot growth but are thought to also have a bio-stimulant effect on plants, increasing resistance to abiotic and biotic stress (Khan et al., 2009). The majority of seaweeds sold commercially are brown algae, specifically *Ascophyllum nodosum* and *Ecklonia maxima* (Craigie, 2011). It has been suggested that seaweed extracts may have some activity against PPN abundance and fecundity primarily in laboratory and greenhouse settings. While the mode of action for such phenomena is largely unknown, the literature suggests that seaweed extracts may be useful to protect plants against biotic stress due to seaweeds possessing unique compounds such as fuciodans and alginates, which may act as elicitors to prime plants for future pathogen defense (Ali et al., 2019).

Integrated pest management (IPM) programmes may be the future of nematode control. They often focus on prevention, as once a nematode population has established it can be very difficult to reduce. Prevention typically involves cultural management of the crop, increasing general plant health and resistance and increasing biodiversity (Stenberg, 2017). Cultural practices employed can include crop rotation, soil aeration and the growing of nematode-resistant cultivars among others (Katan, 2000). Improving plant health by increasing nutrition through fertilizer use may better equip a plant to defend itself against pathogens and pests. This is where frequent applications of seaweed extracts could be useful to increase root length and ensure the plant can have access to a higher nutrient availability. Biological amendments, such as arbuscular mycorrhizal fungi and biosimulants, can also be used to enhance plant health by providing the plant with access to more nutrients, and enabling greater tolerance to pest and disease attack through enhanced growth or increased defences (Viane et al., 2013). While these biostimulants and biological methods may be useful for plant defences by producing a healthier plant, there are two nematode-specific plant protection products on the market, fluopyram and garlic extracts, which can more readily control nematode populations. However the use of these active ingredients can be problematic, for example fluopyram has been labeled as an environmental hazard, and garlic extracts have also been shown to kill to beneficial mycorrhizal fungi in the soil (Baylis, 2019).

To date, it is unclear if there is a general pattern of seaweed products having a detrimental effect on plant parasitic nematode populations. Such knowledge could be of great use in the development of future integrated pest management methods for these pests and could represent part of a sustainable, more natural control method than synthetic chemicals. Furthermore, in order to obtain reliable and reproducible results in the field it is important to identify the laboratory and greenhouse practices which may have increased or decreased the success of using seaweed extracts against PPNs. The use of meta-analysis allows for comparison between the effect sizes of these different studies, whilst simultaneously retaining the variability and uncertainty from the original datasets to allow for an overall effect size to be calculated (Mezeli et al., 2020). This is fundamental when assessing matters within the soil as much is unknown and the complexity of trophic and community interactions mean identifying influential co-factors within the constraints of an applied experiment is difficult. Through the use of meta-analysis these co-factors may be identified and future applied research in the field could have higher success rates, as often it is hard to translate positive PPN laboratory results into a field setting (Martin et al., 2007).

To assess how the use of seaweeds may affect RKN populations and which additional co-factors may be important, our aim was to use meta-analysis of appropriate literature to determine if seaweed extracts could have potential as ‘control’ agents for nematode pests. Our objectives were to answer the following questions: 1) Do seaweed extracts reduce RKN abundance? 2) If effective, do seaweeds work against all RKN species? 3) Do different types of seaweed species have differential population effects? 4) Can seaweed reduce RKN fecundity and egg hatching? and 5) Are these effects seen across laboratory, greenhouse and field studies? We hypothesized that seaweed extracts would have negative effects on RKN performance, but that these would be dependent on the seaweed species used, the preparation method of the extract and the experimental conditions (whether highly controlled or in the field). By taking a holistic approach to the analysis, we hope to identify research needs that will inform future control strategies of nematode pests.

## 2 Materials and methods

### 2.1 Literature search

The Web of Science (ISI) electronic database was searched for the time period 1950 to 2020, using the ‘All databases’ filter. The search terms were nematode AND seaweed, plant parasitic nematode AND seaweed, seaweed AND each of nine different genera of PPN and seaweed AND each of four different genera of seaweed. The nine different PPN genera were *Meloidogyne, Pratylenchus, Rotylenchus, Globodera, Radophulus, Ditylenchus, Xiphenema, Tylenchulus* and *Helicotylenchus*. These PPN were selected as they are either the most economically damaging of crops from different regions, or, the most frequently observed in the field in UK soils (Jones et al., 2013). The different seaweed genera searched for were those commonly sold commercially (Khan et al., 2009), namely *Ascophyllum*, *Ecklonia*, *Durvilea* and *Macrocystis*. Of note, cyanobacteria (blue/green algae) were not accepted as seaweed. Publications including non-PPN nematodes were excluded.

A total of 28 publications were found that fitted these initial search criteria. Unpublished data from our laboratory was also added to avoid bias within the publication of mostly significant results (Koricheva et al., 2013). One paper was excluded for having variances of zero. Publications with no standard deviations/standard errors were omitted if the author was not able to provide such data. This was a common occurrence, many of the papers were from the 1980s and 1990s and it is likely that non-published data from these studies is not retrievable.

### 2.2 Data collation and extraction

Of the 28 publications only 12 could be used in the final analysis, producing a total of 142 separate experimental studies. The 12 publications meet the criteria outlined above and sufficiently provided standard deviations/standard errors. The publications used included: Featonby-Smith and Van Staden (1990); Whapham et al. (1994); Wu et al. (1997); Jenkins et al. (1998); Wu et al. (1998); Manilal et al. (2009); Malusa et al. (2012); Ngala et al. (2016); Radwan et al. (2012); El-Ansary and Hamouda (2014); Laquale et al. (2018) and D'Addabbo et al. (2019).

When means and standard deviations were unavailable in a table, they were digitized from graphs using Web Plot Digitiser v.4.2 (Rohatgi, 2020). Means and standard deviations/standard errors were extracted on nematode hatching rate, nematode abundance, number of galls and eggs/egg masses, nematode activity and number of females/developmental stages. Other parameters included nematode activity during in vitro experiments where nematode motility and infectivity were assessed in various motility tests. All of these parameters were used in the final analysis. Although the starting point for the analysis was open to eight/nine PPN genera, the remaining papers following filtering were all on *Meloidogyne* spp. The majority of publications were also on *A. nodosum*, possibly due to it being the most commercialized.

### 2.3 Statistical analysis

The majority of publications made multiple observations. For example, one paper measured nematode number, hatching, infectivity/attraction and orientation (Ngala et al., 2016). In these cases, each measurement was recorded in the analysis as a separate parameter, since it is most unusual for all life history parameters of any organism to respond in the same way (Koricheva et al., 2013). Unless stated otherwise all analyses were carried out using the OpenMEE interface (Wallace et al., 2017) of the ‘metafor’ package in R 3.6.0. A random effects model using standardised mean difference, Hedges (*d)*, was used to calculate overall effect size (grand mean) and heterogeneity (tau^2^). A random effects model was used to account for differences in treatment effects and experimental conditions variability. The following subgroup analysis was also carried out using the Hedges (*d*) random effect model. Separate sub-group meta-analyses were conducted on the following co-factors: seaweed species, seaweed extraction, crop (plant), seaweed dosage, experimental conditions (laboratory, greenhouse, field), application type (liquid/granular) and nematode species. Specific sub-group analysis was carried out on the effects of *A. nodosum* on nematode species and nematode parameters. As our analysis was based on a relatively small number of papers, study was included as a random factor in the models, to account for any potential bias.

A mixed effects meta-regression model was used to test for pairwise differences between categories, to further understand how nematode abundance/performance was affected by the moderator variables. Between group heterogeneity (Qm) was used to test for moderator significance, where a significant Qm indicated differences in mean effect size between moderator levels (Gange et al., 2019). Experiments where *n* < 2 were omitted from meta-regression analysis, as were experiments that had “not_specified” parameters.

The dataset was examined for publication bias, via three methods: the failsafe N was calculated using both the Rosenberg and Rosenthal method. The failsafe N was calculated to address publication bias and ascertain how many insignificant studies would be needed to produce a non-significant result. The failsafe Rosenberg number was 1575, which is larger than 5 N + 10 (720). The failsafe N Rosenthal number was 4846 and although this metric is sensitive to the number of unpublished studies, these were relatively few in number in our database. Funnel plots were also made using R package metafor, again to check for publication bias, and are presented in the Supplementary on-line material. Overall, these analyses indicate that our results are robust against possible publication bias. All data are available at doi:10.17637/rh.15022488.v1

## 3 Results

The mean effect size for the whole dataset was significantly negative (Hedges *d* *=* -0.522, CI = (-0.708, -0.337), *P* < 0.001) suggesting that the use of seaweed extracts can reduce nematode performance. The results showed moderate heterogeneity (Meta regression, *Q* = 437.410, d.f. = 141, *i*^2^ = 56.54), so the following analysis was performed using a random-effects model.

**Fig. 1:**
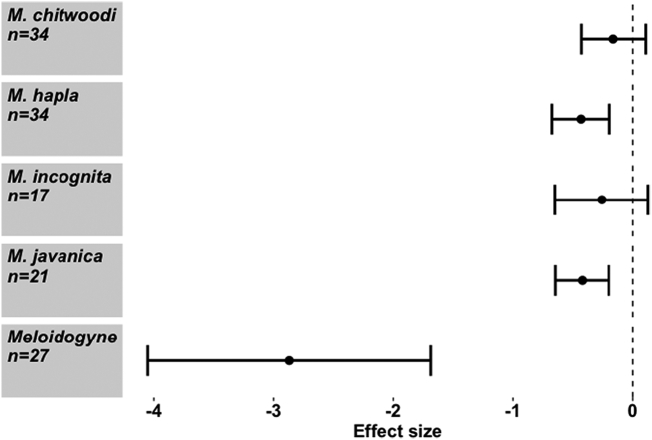
Effect of seaweed don mon different *Meloidogyne* spp. performance. Values are mean effect size (Hedges’ *d*) and negative values mean that seaweed has an antagonistic effect on that species. Error bars represent 95% confidence intervals, and effect size is considered significant when these do not overlap zero (vertical line). *n* denotes number of effect sizes for each nematode species.

**Fig. 2:**
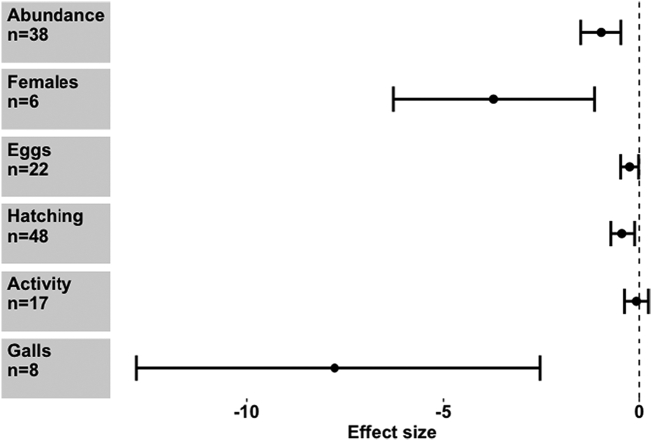
Effect of seaweeds on different nematode performance parameters. Values are mean effect size (Hedges’ *d*) and negative values mean that seaweed has an antagonistic effect on that parameter. Error bars represent 95% confidence intervals, and effect size is considered significant when these do not overlap zero (vertical line). *n* denotes number of effect sizes for each nematode parameter.

**Fig. 3:**
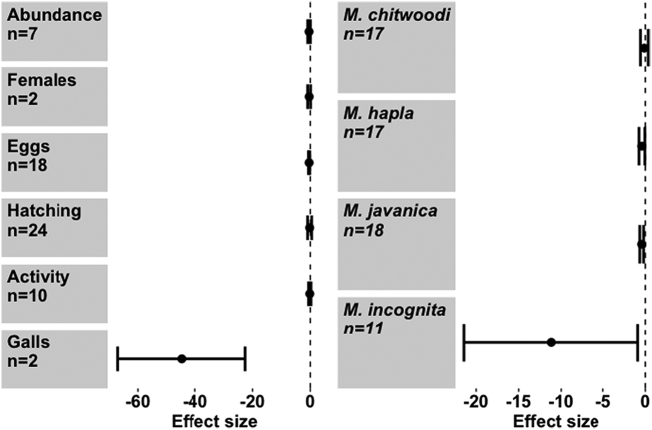
Effect of *Ascophyllum nodosum* extracts a) on different *Meloiodgyne* species and. b) on *Meloidogyne spp* life stages. Values are mean effect size (Hedges’ *d*) and negative values mean that *A. nodosum* has an antagonistic effect on that species or life stage parameter. Error bars represent 95% confidence intervals, and effect size is considered significant when these do not overlap zero (vertical line). *n* denotes number of effect sizes for each nematode species or parameter.

**Fig. 4:**
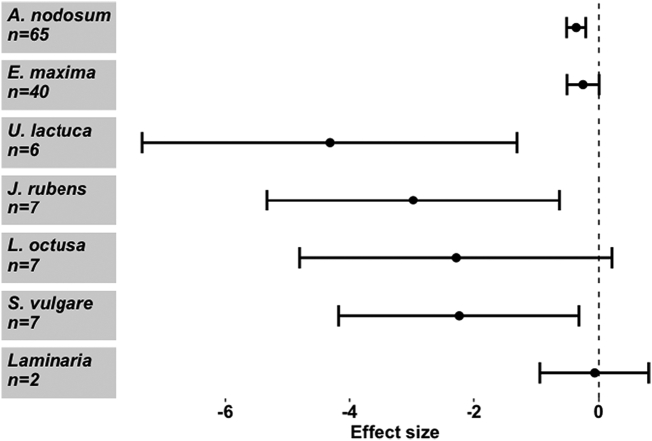
Effect of different seaweed species on nematode performance. Values are mean effect size (Hedges’ *d*) and negative values mean that the seaweed species has an antagonistic effect on PPNs. Error bars represent 95% confidence intervals, and effect size is considered significant when these do not overlap zero (vertical line). *n* denotes number of effect sizes for each seaweed species.

When considering the effect of seaweed on different PPN species, *M. hapla* and *M. javanica* appeared to be more adversely affected by the seaweed extracts than *M. chitwoodi* or *M. incognita* (Meta regression, *Q*_m_ = 35.76, d.f. = 4), *P* < 0.001) (Fig. 1). Seaweed also exerted a strong negative effect on various stages of the *Meloidogyne* life cycle, though to differing extents (Meta regression, *Q*_m_ = 34.61, d.f. = 6), *P* < 0.001). Parameters negatively affected were overall abundance, female numbers, egg production, hatching rate and gall number (Fig. 2). PPN movement (activity) was not significantly reduced. Further sub-group analysis of the effect of *A. nodosum* on *Meloidogyne* spp. shows that *M. chitwoodi* was again unaffected, while *M. hapla, M. javanica* and *M. incognita* were negatively affected (Fig. 3a). *Ascophyllum nodosum* exerted a negative effect on egg production and gall number only (Meta regression, *Q*_m_ = 23.7, d.f. = 5), *P* < 0.001), though sample sizes were relatively small (Fig. 3b). *Ascophyllum nodosum* exerts a highly significant negative effect on RKN (Hedges *d* = -0.362, CI = (-0.515 - -0.21) *P* < 0.001, *n* = 65). This was a stronger effect than that of *E. maxima* (Hedges *d* = -0.254, CI = (-0.51–0.003), *P* = 0.053, *n* = 40) which is also commonplace in the seaweed market. The other seaweeds rarely found commercially (Craigie, 2011), that had a significant negative impact on RKN, were *Ulva lactuca*, *Jania rubens* and *Sargassum vulgare*, but overall, effects differed from one species to another (Meta regression, *Q*_m_ = 39.07, d.f. = 5), *P* < 0.001) (Fig. 4). While *U. lactuca, J. rubens* and *S. vulgare* tended to show large negative effect sizes, the samples sizes for these species were small, and confidence intervals large, compared with *A. nodosum* and *E. maxima*.

**Fig. 5:**
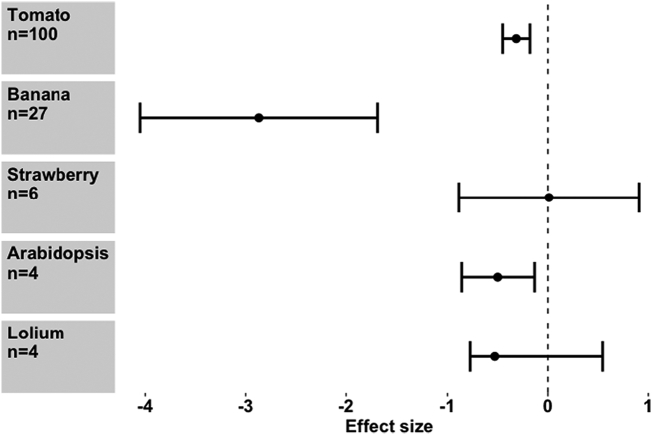
Effect size seaweed extract application on different crops. Values are mean effect size (Hedges’ *d*) and negative values mean that seaweed has an antagonistic effect on nematodes attacking that crop species. Error bars represent 95% confidence intervals, and effect size is considered significant when these do not overlap zero (vertical line). *n* denotes number of effect sizes for each crop species.

**Fig. 6:**
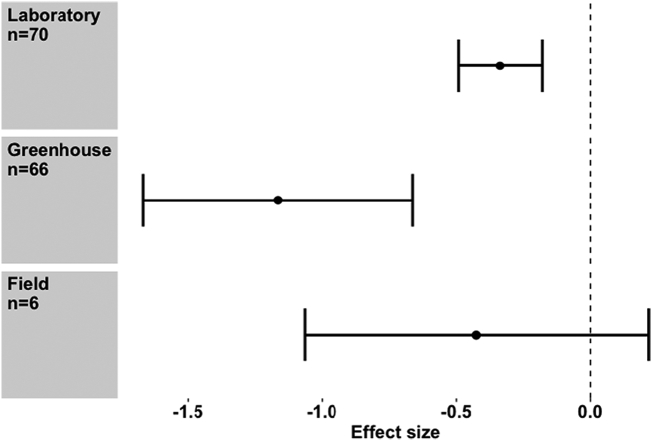
Effect of seaweed application on root knot nematode performance in different experimental settings. Values are mean effect size (Hedges’ *d*) and negative values mean that seaweed has an antagonistic effect on nematodes in that environment. Error bars represent 95% confidence intervals, and effect size is considered significant when these do not overlap zero (vertical line). *n* denotes number of effect sizes for each experimental location.

The identity of the host plant appears to be important, in that experiments carried out on *Solanum lycopersicum* (tomato), *Musa* spp. (banana) and *Arabidopsis thaliana* show a significant negative effect on *Meloidogyne* spp., whereas *Fragaria* (strawberry) and *Lolium perenne* (perennial ryegrass) did not show a significant effect (Meta regression, *Q*_m_ = 36.31, d.f. = 4), *P* < 0.001) (Fig. 5). Meanwhile, the location of the experiment was also important (Fig. 6). In the laboratory and greenhouse settings there was a strong negative reduction in PPN performance (Hedges *d* = -0.335, *p* ≤0.001, *n* = 70; d = -1.196, p ≤0.001, *n* = 64 respectively), whereas the reduction in PPN was not significant in the field. However, the difference between moderators was weak (Meta regression, *Q*_m_ = 4.95, d.f. = 2, *P* = 0.08), most likely because the number of field studies was still relatively small, comprising of 6 separate field studies (Fig. 6). A wide range of seaweed dosage rates was used by authors but there were no differences between them (Meta regression, *Q*_m_ = 14.09, d.f. = 9, *P* < 0.05).

## 4 Discussion

While previous studies may have demonstrated that seaweed has an adverse effect on RKN in the laboratory and greenhouse, we believe that this is the first study that demonstrates that some seaweed species are more useful than others when managing RKN. This study also highlights the different RKN life cycle stages that are affected by seaweed extract applications. The findings show that seaweed extracts can reduce several parameters of PPN performance, with particular effects on the abundance of the root knot species *M. javanica* and *M. hapla*, although specific seaweed species adversely affect *M. incognita*. The population declines of the *Meloidogyne* spp. occurred across all life cycle stages measured, and included reduced hatching and egg numbers, reduced J2 populations and female numbers and reduced galling. Further group analysis showed that *A. nodosum* extracts can also reduce *M. incognita* abundance. A number of conditions were identified that may affect the impact of seaweed extracts on PPN populations; these included the species of seaweed used, the host crop, the *Meloidogyne* species being targeted and their life cycle stage. *Ascophyllum nodosum* appeared to have the most detrimental effect on *Meloidogyne* populations compared to other seaweed species, such as *E. maxima*. Seaweed reduced *Meloidogyne* populations when they were associated with a tomato, *Arabidopsis* and banana rhizospheres, however this was not observed in strawberry crops or on perennial rye grass used in sports turf.

Previous publications have generated conflicting results when measuring RKN fecundity after seaweed application (Whapham et al., 1994; Wu et al., 1998; Ngala et al., 2016). Some authors report finding a seaweed-triggered reduction in egg retrieval and hatching rate, whereas others have failed to replicate this. Despite previous opposing findings, this study found seaweed use significantly reduced hatching rate and egg number. This reduction in hatching rate was only slightly more significant than the reduction in egg retrieval. The overall RKN abundance and galling was most significantly decreased. While conjecture, it could be reasonable to assume that a lower hatching rate could be the reason for lower J2 and adult abundances and hence lower visible galling. As varying dosage rates seemed to be of very little consequence to RKN populations, it may not be dosage rates, but application timing, that could be the most important factor to consider, particularly as the half-life of seaweed extracts is unknown. Timing applications before hatching could reduce hatching and subsequent population numbers and visual disease symptoms. However as these results have not been replicated in the field more field research is needed.

This study demonstrates that seaweeds can reduce *Meloidogyne* numbers, although some species are more affected than others. Overall, an application of seaweed resulted in the largest population reduction of *M. javanica* and *M. hapla*, however the only seaweed species to reduce *M. incognita* abundance was *A. nodosum, M. chitwoodi* showed no significant change in abundance. This apparent species-specific effect elicited by seaweed has previously been observed in the literature; Wu et al. (1997) found strong reductions in *M. incognita* numbers compared to *M. javanica* after *A. nodosum* extract application and, similar results were found by Jenkins et al. (1998). This study further suggests that *A. nodosum* extract may be particularly useful when trying to reduce *M. incognita* populations.

It also seems that it is not only nematode species that may influence seaweed efficacy but also the crop species to which the seaweed is applied. The majority of the research in this area has been conducted on tomato crops, meaning tomato had the largest replication number in this study and subsequently the strongest negative results. Strawberry crops and turf grass had smaller replicate numbers and did not yield significant results. While this may suggest that some effect sizes are a consequence of replicate numbers, banana and *Arabidopsis* had much smaller sample sizes, but still showed strong negative results, suggesting that this was a genuine biological effect of seaweed on nematodes in these plant species. It has been well known for some time that some crops are more resistant to RKN, while others are more susceptible (Maleita et al., 2012). For example many cultivars of tomato appear to be susceptible to *Meloidogyne spp*, whereas many strawberry cultivars have been deemed as poor hosts by some (Freitas et al., 2017). There may be differences in RKN host selection depending not only on the host and RKN species but also the nematode strain and plant cultivar, meaning it could be far more species-specific then once thought. This may be due in part, to the production, or lack of, nematode repelling volatile organic compounds (VOCs) or a plants ability to adequately respond to PPN attack via mechanisms such as reallocating resources to shoots, producing defensive phytohormones such as jasmonic acid and/or increasing root strength (Da Silva et al., 2019). The non-significant result seen in ryegrass could be attributed to the common of occurrence of sports turf trials to produce small but non-significant reductions in RKN number after seaweed application (Tarjan and Frederick, 1983; Sun et al., 1997). Despite conflicting evidence in turf grass systems, many other plant species (banana, tomato, *Arabidopsis*) were shown by this study to exhibit a seaweed-induced reduction in RKN, possibly as a result of the crop species, cultivars and *Meloidogyne* strain used in these studies.

Factors such as algal species, season, location of the raw seaweed and the extraction process can change the chemical composition of the seaweed extract and alter its efficacy (Sharma et al., 2012; Khairy and El-Shafay, 2013). The majority of the seaweeds used in the meta analysis were brown alga (*A. nodosum, E. maxima, S. vulgare, Laminaria*), however a red *(J. rubens*) and a green (*U. lactuca*) alga were also included in this study. Both the red and the green alga showed significant effect sizes, as did the majority of the brown alga. These results are interesting, as different seaweed extracts will have different compound profiles. For example, brown alga has a unique metabolism when compared to green alga as it produces more mannitol instead of sucrose resulting in extracts that may have high amounts of mannitol and sugar alcohols (Belghit et al., 2017). Red and green algae may contain more fucoidans when compared to brown alga (Olsson et al., 2020). While the exact mode of action of these compounds (such as fucoidans) is unknown, they may produce a ‘bio-stimulant’ like effect in plants and increase resistance to pathogens and abiotic stressors (Vera et al., 2011). This may explain why *A. nodosum* is the most effective at reducing nematode populations. Perhaps it has the highest amount of “elicitor-like” compounds in comparison to *E. maxima.* Species such as *M. incognita* may only be affected by higher abundances of particular compounds found in *A. nodosum* extracts.

Previous literature suggests that alkali extractions often used when processing *A. nodosum* may separate alginate and auxins more readily and the use of high temperatures may produce more fucoidans in a seaweed extract (Silva et al., 2015). *A. nodosum* extracts in this study were exclusively processed using an alkaline extraction, whereas *E. maxima* extracts were processed using cell-burst technology. Therefore it is impossible to say whether differences from extracts were a result of seaweed species or the extraction processed used.

Surprisingly, some of the literature suggests that seaweed extracts caused immobility in *M. incognita*, *M. javanica* and *M. acrita* (to a lesser extent) in motility tests, implying seaweed could have nematicidal properties (Paracer et al., 1987; Rizvi and Shameel, 2006; Nour El-deen and Issa, 2016; Ghareeb et al., 2019). However, the results of the meta analysis showed that no effect on RKN activity during motility and attraction tests, and, perhaps ‘nematicidal’ effects previously found are due to solvents being used in the extracts; or, due to the use of extremely high concentrations of seaweeds in vitro (>80%), such high rates would normally not be used in the field.

The precise mode of action of seaweed on nematodes is largely unknown although several authors have speculated as to why it may reduce PPN numbers. The presences of betaines found in alkaline processed *A. nodosum* extracts have been considered as contributing to RKN reduction (Wu et al., 1997: Wu et al., 1998), cytokinins may also play an important role (Featonby-Smith and Van Staden, 1983). It has also been suggested that seaweed application may stimulate plant formaldehyde production causing an increase in host plant resistance (Jenkins et al., 1998). The theory that seaweeds may increase plant resistance to pathogens and pests is a common thread in many publications and has recently been demonstrated in *Arabidopsis* where an application of *A. nodosum* triggered the expression of genes associated with plant defense responses i.e. *PR-1* expression, subsequently reducing pathogenic bacteria colony forming units (CFUs) associated with plant tissue (Cook et al., 2018).

Often it is difficult to replicate PPN experimental findings from the laboratory and greenhouse with those from the field (Crouch and Van Staden, 1993; Martin et al., 2007). The analysis between laboratory, greenhouse and field trials confirmed this and revealed examples where laboratory and greenhouse experiments were significant but field trials were not. This may be due to the high complexity of soil trophic interactions in the field, however it could also be due to application timing. Perhaps soil analysis needs to be carried out to determine RKN life cycle stage, in order to reduce hatching. Also in soils with high drainage such as on sports turf pitches, the seaweed might be simply being flushed through. In most of the laboratory studies the nematodes are immersed in the seaweed extract, perhaps a soil drench instead of a spray application would be better suited for this purpose. Field trials are few in number and it is possible that many non-significant results may not have been published. Future research could look at conducting applied field experiments to determine if seaweed extracts have a genuine potential to reduce PPN infection incidence in the field.

While more research into the use of seaweeds against nematodes in the field is required, the results from laboratories and greenhouses seem promising. The results suggest that perhaps seaweeds could be integrated into IPM schemes for PPN prevention pre-planting. Further research is needed to determine if subsequent applications of seaweed would be necessary to keep populations at a manageable size. While current control methods involve the use of chemicals such as garlic extract or fluopyram, we propose that seaweed extracts could be used in a similar manner and data from the laboratory trials included in this analysis suggest that these may work curatively as well as preventatively. After nematode testing is used to ascertain the RKN life cycle stage, seaweed application could then be timed to reduce hatching rate, with applications being made when the majority of nematodes are most likely in the egg laying or hatching phase of their life cycle. Future research will also need to determine appropriate volumes of seaweed for field applications. In some of the laboratory studies, RKN eggs were submerged in seaweed extract solutions, which could be difficult to recreate in a field setting.

## 5 Conclusions

This meta analysis does give indications that seaweed extracts (particularly *A. nodosum*) could indeed be used to manage RKN infections, perhaps providing cheaper methods of *Meloidogyne* management when compared with other methods, such as biofumigation (see e.g. Peiris et al., 2020; Waisen et al., 2020). The mode of action is unknown, but this poses an interesting question for future research. The results of this study serve as a promising start into seaweed use beyond just that of a biostimulant. Following further research, perhaps seaweeds could be used in integrated pest management schemes on a range of different crops not only as a soil conditioner, but also to reduce RKN damage. As seaweed can be harvested sustainably, it is safe to use and has no known adverse effects on the environment, making it an interesting candidate for PPN control. This is especially the case when considering the loss of nematicides from the pesticide market.

The following is the supplementary data related to this article.

Supplementary on-line material Funnel plot to further investigate publication bias of the studies used in the meta analysis. Each dot represents each separate study; the standard error is used as a measure of study precision. While the plot may show an asymmetric distribution, which could suggest publication bias, the other measures used (Rosenthal and Rosenberg numbers) did not suggest publication bias. Supplementary on-line material

## Declaration of competing interest

The authors declare that they have no known competing financial interests or personal relationships that could have appeared to influence the work reported in this paper.
