# Supplementary material for: Evaluating the use of seaweed extracts against root knot nematodes: A meta-analytic approach
Source: Appl Soil Ecol. 2021 Dec;168:None. doi: 10.1016/j.apsoil.2021.104170 (PMC8501307; doi:10.1016/j.apsoil.2021.104170)
Supplement: Supplementary on-line material — Funnel plot to further investigate publication bias of the studies used in the meta analysis. Each dot represents each separate study; the standard error is used as a measure of study precision. While the plot may show an asymmetric distribution, which could suggest publication bias, the other measures used (Rosenthal and Rosenberg numbers) did not suggest publication bias. [file mmc1.docx]

liamSupplementary materials


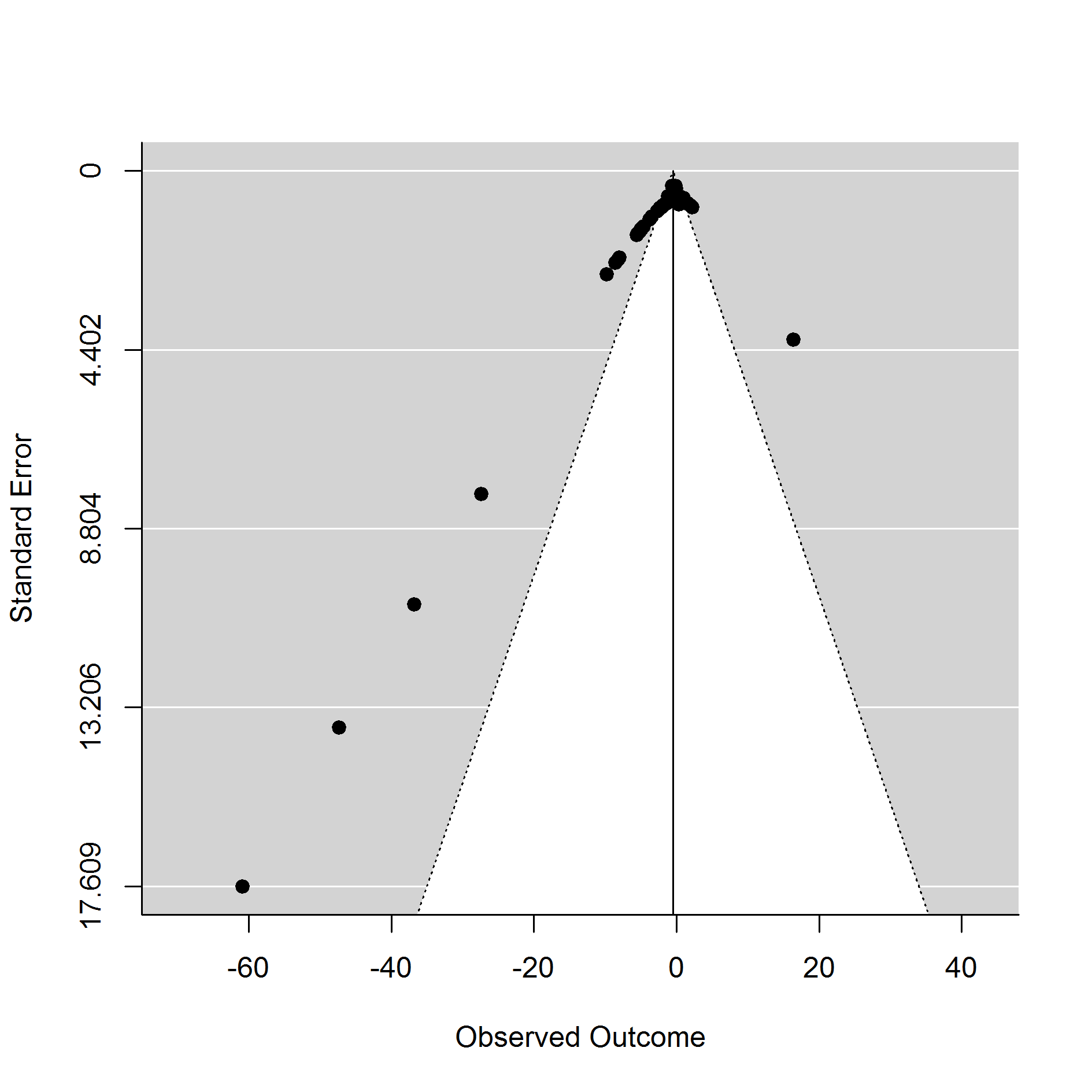


Figure 1 - Funnel plot to further investigate publication bias of the studies used in the meta analysis. Each dot represents each separate study; the standard error is used as a measure of study precision. While the plot may show an asymmetric distribution, which could suggest publication bias, the other measures used (Rosenthal and Rosenberg numbers) did not suggest publication bias.
